# Pin1 Modulation in Physiological Status and Neurodegeneration. Any Contribution to the Pathogenesis of Type 3 Diabetes?

**DOI:** 10.3390/ijms19082319

**Published:** 2018-08-08

**Authors:** Marzia Bianchi, Melania Manco

**Affiliations:** Research Unit for Multi-factorial Diseases, Obesity and Diabetes, Bambino Gesù Children’s Hospital IRCCS (Istituto di Ricovero e Cura a Carattere Scientifico), viale di San Paolo 15, 00146 Rome, Italy; marzia.bianchi@opbg.net

**Keywords:** Alzheimer’s disease, brain glucose metabolism, neuronal differentiation, neuronal degeneration, Prolyl isomerases, Pin1, type 2 diabetes, type 3 diabetes

## Abstract

Prolyl isomerases (Peptidylprolyl isomerase, PPIases) are enzymes that catalyze the isomerization between the *cis*/*trans* Pro conformations. Three subclasses belong to the class: FKBP (FK506 binding protein family), Cyclophilin and Parvulin family (Pin1 and Par14). Among Prolyl isomerases, Pin1 presents as distinctive feature, the ability of binding to the motif pSer/pThr-Pro that is phosphorylated by kinases. Modulation of Pin1 is implicated in cellular processes such as mitosis, differentiation and metabolism: The enzyme is dysregulated in many diverse pathological conditions, i.e., cancer progression, neurodegenerative (i.e., Alzheimer’s diseases, AD) and metabolic disorders (i.e., type 2 diabetes, T2D). Indeed, Pin1 KO mice develop a complex phenotype of premature aging, cognitive impairment in elderly mice and neuronal degeneration resembling that of the AD in humans. In addition, since the molecule modulates glucose homeostasis in the brain and peripherally, Pin1 KO mice are resistant to diet-induced obesity, insulin resistance, peripheral glucose intolerance and diabetic vascular dysfunction. In this review, we revise first critically the role of Pin1 in neuronal development and differentiation and then focus on the in vivo studies that demonstrate its pivotal role in neurodegenerative processes and glucose homeostasis. We discuss evidence that enables us to speculate about the role of Pin1 as molecular link in the pathogenesis of type 3 diabetes i.e., the clinical association of dementia/AD and T2D.

## 1. Introduction

Prolyl isomerases (Peptidylprolyl isomerase, PPIases) are a class of enzymes that catalyze the *cis*/*trans* isomerization of the peptide bond between the preceding amino acid and the proline residue (Pro) [1,2,3]. The presence of Pro’s unusual structure lowers the free energy difference and allows these conformational changes [4]. The catalytic activity of PPIases modulates enzyme activity, protein stability and cellular localization by mediating conformational changes of substrates [3,5,6,7,8,9]. Three distinctive groups belong to the class of PPIases: the FKBP (FK506 binding protein), the Cyclophilin and the Parvulin families (Peptidylprolyl *cis*/*trans* isomerase NIMA-interacting 1 (Pin1) and Par14) [1,3,7,8]. 

With respect to other PPIases, the distinctive feature of Pin1 is that the Serine (Ser) or the Threonine (Thr) that precedes the Pro residue is phosphorylated accounting for the correct enzyme-substrate recognition [3,9]. The proline-directed kinases phosphorylate Pin1 substrates and induce Pin1-dependent post-phosphorylation conformational changes that impact key proteins. These changes influence cellular functions such as mitosis, differentiation and metabolism. Deregulation of Pin1 substrates has been implied in the onset of various diseases, i.e., cancer, neurodegenerative disorders and metabolic syndromes including type 2 diabetes (T2D) [2,3,4,10,11,12].

Expression of Pin1 increases significantly in cell types that undergo active cell division [4,13], depending on cell type, availability of specific substrates, and biological context. Pin1 seems to play a dual role, promoting cell proliferation or even, at the opposite, cell death depending on the biological context. Different capacities result from the adoption of diverse mechanisms of regulation, i.e., gene transcription and protein phosphorylation. 

Pin1 serves as molecular “switch” that modulates enzyme activity or an entire signaling pathway [14]. Deeper understanding of function and regulation of Pin1 is essential to appreciate fine mechanisms of signal transduction and integration that are triggered by the propyl isomerase. 

In this review, we will discuss the pivotal role of Pin1 in physiological i.e., the neuronal development as well as in neurodegenerative conditions i.e., the Huntinton’s, the Parkinson’s, the temporal lobe epilepsy (TLE) and the Alzheimer’s (AD) diseases. 

Then, with regard to AD, important features of the disease phenotype are reduced brain utilization of glucose and impaired glycolytic enzyme activities owing to defective insulin signaling [15]. Therefore AD is deemed as “type 3 diabetes” (T3D) [16] and regarded a metabolic disease. de la Monte defines AD “as a brain form of diabetes in which insulin resistance and deficiency develop either primarily in the brain, or due to systemic insulin resistance disease with secondary involvement of the brain” [17]. Interestingly, in patients with AD, Pin1 seems involved not only in neuronal degeneration but also in brain impaired glucose metabolism. Therefore, Pin1 may be at the crossroad between AD and T2D-associated dementia, contributing to the clinical relationship of these two conditions in the T3D.

## 2. Pin1 Characterization: Structure, Regulation and Subcellular Localization

Pin1 is a ubiquitous enzyme, with high similarity across species (*Homo sapiens*, *Xenopus laevis*, and *Danio rerio*) that suggests conserved function in vertebrates [4]. X-ray crystallography and solution nuclear magnetic resonance (NMR) studies in extracts of human brain tissues show that Pin1 is a monomeric enzyme of 163 amino acids. Pin1 consists of two functional domains, the WW- at the N-terminus and the PPIase-domains at the C-terminus, covalently fastened by a flexible linker (amino acids 38–53) of 15-residues [9,18]. The linker allows the domains to rotate independently of each other [19,20]. The PPIase domain catalyzes specifically the *cis*/*trans* isomerization of pSer/pThr-peptidyl-prolyl bonds. The WW domain (type IV) binds, but does not catalyze, similar epitopes [20,21]. Upon Pin1 binding of the specific substrate to the previous phosphorylated S/T-P motifs by proline directed kinases, the two functional domains of Pin1 interact partially with each other in a substrate-dependent manner [20,22,23]. Recognition of the substrate determines a loss of flexibility of residue side-chains in the region between the catalytic loop and the inter-domain surface. The reduced flexibility leads to increased contacts between the two functional domains [23].

Both E2F (E2 factor) [24], a protein involved in the cell cycle regulation, and N1ICD (Notch Intracellular Domain) [25], a protein that regulates Pin1 generating a positive loop in breast cancer cells, are able to activate transcription of Pin1 upon binding. On the contrary, AP4 (Activating Enhancer Binding Protein 4), the brain-selective transcription factor [26], down regulates Pin1 transcription, and the proteasome is responsible of the protein degradation following its ubiquitination [4,27]. 

Protein kinases regulate the capability of Pin1 to bind substrates. Steps for binding are phosphorylation of S/T-P motifs of substrates by kinases first, and secondly, phosphorylation of specific Ser residues in the Pin1 WW domain. Phosphorylation at the Ser16 residue modulates the ability of Pin1 to bind its substrates in a substrate-dependent manner [3]. In particular, phosphorylation by PKA (Protein kinase A) [28] and Aurora A [29] kinases inhibited the binding of Pin1 to its substrate resulting in mitotic block [28] or progression to G2/M phase [29]. Conversely, both Ser/Thr-kinase RSK2 (ribosomal protein S6 kinase 2) [30] and COT (Cancer Osaka Thyroid) [31] increased the binding of Pin1 to substrates leading to tumor progression.

Other kinases induce post-translational modifications of Pin1 in different Ser residues. The phosphorylation on Ser71 by DAPK1 (Death-associated protein kinase 1), a known tumor suppressor, inhibited Pin1 catalytic activity during the cell cycle progression [32]. MLK3 (Mixed Lineage Kinase 3) is a MAP3K (Mitogen-activated protein kinase kinase kinase) phosphorylated Pin1 on Ser138, thus increasing its activity to drive cell cycle progression [33]. Phosphorylation on Ser65 by PLK1 (Polo-like kinase 1), a regulator of mitotic checkpoints, stabilized Pin1 structure, reducing its ubiquitination and consequently degradation [27].

As to the exact subcellular localization of Pin1, this is not unique. Pin1 subcellular localization seems to vary upon post-translational modification of different Ser residues and different cellular contexts. When the Ser16 residue was phosphorylated and Pin1 modulation was associated with mitotic arrest, Pin1 was found in the nucleus [28,29]. When the same residue was phosphorylated in mouse embryonic fibroblasts and breast cancer cells [30,31], Pin1 was detected out of the nucleus. When Pin1 was phosphorylated on Ser138 enhancing tumorigenesis, Pin1 *trans*-located into the nucleus [33].

Subcellular localization of Pin1 seems to vary in different cell types, tissues and health status. Pin1 localized in the nucleus of cell lines such as SH-SY5Y, but also in the cytoplasm of primary neuron cultures or tissues [4]. Pin1 presented a cytoplasmic localization in axons from cultured DRG (dorsal root ganglia) neurons [34], but it was localized in both compartments at comparable levels in primary cultured mouse cortical neurons [35] and embryonic NPCs (neural stem/progenitor cells) [36]. Ibarra and co-workers found that Pin1 was preferentially localized in the cytoplasm of a number of neuronal cells, but there was nuclear localization in some cases or even nuclear exclusion of Pin1 in some other cases (i.e., in embryos and in adult brain of zebrafish) [4]. In brains from autopsies, PIN1 was found to localize in the nucleus of normal neurons, but both in nuclei and cytoplasm in brains from patients suffering frontotemporal dementias (FTD) [37] or AD [38,39,40]. In AD patients, Pin1 localized also exclusively in the cytoplasm of neurons of certain brain regions i.e., the hippocampus [38,39,40]. 

A sound explanation for the varying cell localization of Pin1 is a dynamic regulation of the enzyme in different cell-types, developmental stages and pathological conditions. Such dynamic regulation might depend on phosphorylation of Pin1 and generation of different isovariants [4]. Indeed, Pin1 showed a number of different phosphorylated isovariants [28,29,30,31,32,33] that were detectable combining bi-dimensional (2D) denaturant gel electrophoresis followed by western blotting analysis in the zebrafish model at different developmental stages [4]. 

## 3. Physiological Role of Pin1 in In Vivo Brain Development

There is a bulk of evidence demonstrating the effects of Pin1 on specific substrates and/or signaling pathways in cell lines. On the contrary, in vivo evidence is limited. We focused on studies performed in in vivo models of mouse and zebrafish to highlight the role of Pin1 in neuronal development.

### 3.1. Pin1 Expression during Embryogenesis in the Zebrafish

The expression of Pin1 is tightly regulated during embryogenesis. Levels of expression vary during stages of embryogenesis and across different regions of the embryo [4]. Pin1 mRNA was already detected at 1–2 cell-stages, indicating a maternal heredity. Its levels decreased during the next developmental steps. As to regional distribution, analysis of Pin1 mRNA/protein level and distribution provided inconsistent results. In whole mount in situ hybridization (WISH), mRNA levels of Pin1 were higher in head regions (cerebellum, ventricular zone of the diencephalon and thelencephalon) and lower in trunk and tail regions. Levels of Pin1 protein were not different between trunk and head regions [4] in immunofluorescence assays.

### 3.2. Pin1 Regulates Neuronal Cortical Differentiation: Modulation of the Wnt/β-Catenin Pathway 

**Figure 1:**
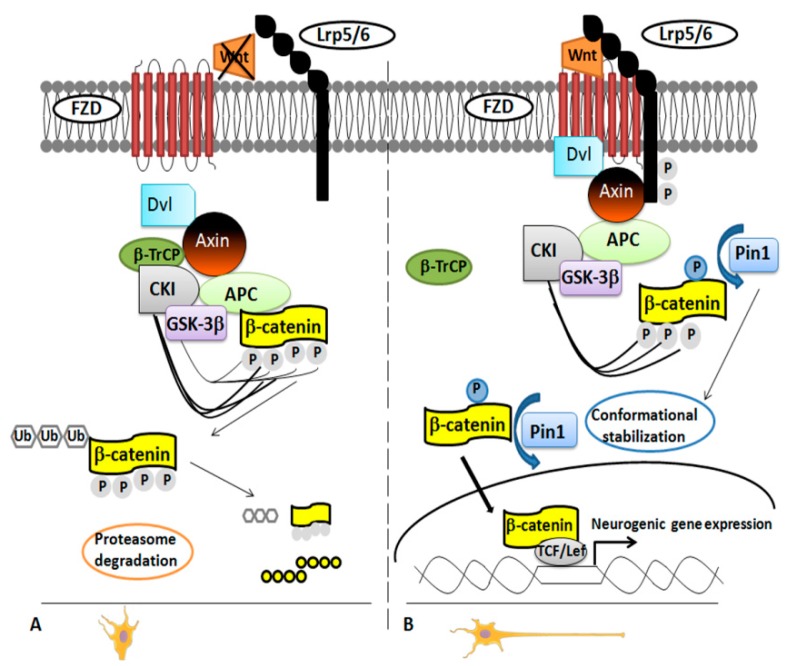
Pin1 modulation of Wnt/β-catenin pathway. **Panel A.** Inactivation of the Wnt/β-catenin pathway. In the absence of Wnt stimulation, levels of β-catenin decrease in the cytoplasm owing to ubiquitination and degradation by the proteasome. Both casein kinase 1α (CK1α) and glycogen synthase kinase-3β (GSK-3β) phosphorylate β-catenin to assemble a complex of proteins (named “destruction complex”). The complex includes scaffold protein axin, adenomatous polyposis coli (APC) and β-transducin repeat-containing E3 ubiquitin protein ligase (β-TrCP). Once phosphorylated, β-catenin is recognized by β-TrCP, ubiquitinated and then degraded. **Panel B.** Activation of the Wnt/β-catenin pathway. A Wnt ligand binds a Frizzled (Fz) receptor and coreceptors LRP5/6 activating the protein Dishevelled (Dvl) mostly by phosphorylation. This modification triggers the recruitment of axin to the phosphorylated tail of LRP and leads to the inhibition of the degradation pathway. Pin1 binds the β-catenin phosphorylated on the Ser246-Pro motif thus downregulating its binding with APC. By doing so, Pin1 catalyzes conformational stabilization of cytoplasmic β-catenin, which gets into the nucleus to regulate the transcription of Wnt target genes.

In 2012, it was demonstrated that Pin1 regulates differentiation of cortical neurons by affecting the Wnt/β-catenin pathway [36]. Pivotal in neurogenesis [36,41], the Wnt/β-catenin pathway works on the two main steps of the neural stem/progenitor cells (NPCs) development: the expansion phase, stimulating proliferation; and the neurogenic phase, regulating the timing and the area of the neuronal differentiation [36,42]. There was no doubt that the β-catenin functionality is regulated by phosphorylation, but the exact mechanism was unclear for long time until β-catenin was identified as specific substrate of Pin1 in NPCs. The researchers used a proteomic approach, glutathione S-transferase (GST)-pull down strategies followed by Electrospray Ionization-Mass Spectrometry (ESI-MS) analysis [36] to demonstrate that Pin1 regulates β-catenin functionality by modulating its conformation after the phosphorylation in the Ser246-Pro motif [36,43]. This occurred in the later phases of neuronal differentiation both in NPCs and mouse brain (Figure 1). 

In NPCs and embryonal mouse cerebral cortex, Pin1 and β-catenin co-localized in both cytosol and nuclei [36].

In Pin1 KO NPCs from embryonal and adult brain, the percentage of differentiated neurons was significantly lower than in WT NPCs [36,44] as evident by using specific markers for neurons and glia cells [36]. Pin1 deficiency did not affect the NPCs proliferation in BrdU assay experiments, but it inhibited specifically cell differentiation into migrating immature neurons at embryonal stage E15.5. These results were consistent with the higher level of Pin1 expression in NPCs at later developmental step. Consistent with this finding, Pin1 KO mice showed an impaired motor activity during the neonatal stage, and this was the result of a specific inhibition of the differentiation of the upper layers neurons in the motor cortex. The authors performed experiments to stimulate/inhibit the signaling in order to confirm the role of Pin1 in the regulation of the Wnt/β-catenin pathway. They observed a rescue of Pin1 KO NPCs phenotype by using constitutively active S33Y β-catenin mutant or NPCs overexpressing Pin1. On the contrary, the truncated TCF4 (DN-TCF4), a dominant-negative mutant of the β-catenin activity, blocked the signaling and resulted in reduced neuronal differentiation [36].

### 3.3. Pin1 Regulation of Axonal Guidance by Modulation of Microtubule Assembly and Buffering Sema-3A Stimulation

During development of the CNS (central nervous system), axonal growth is tightly regulated by many extracellular mediators that are secreted or bound to cell membranes. The binding of these molecules to their receptors at the active growth cones triggers signaling cascades that modify dynamics of microtubules and result in axonal growth, turn, stop, or retraction [34,45]. Mechanisms of the signaling cascade are not fully understood.

Collapsin response mediator protein 2 (CRMP2), a tubulin heterodimer-binding protein that supports microtubule assembly and axon growth, plays a pivotal role in the cascade [46,47,48]. The binding affinity of CRMP2 to tubulin regulates the dynamic equilibrium of microtubule assembly-disassembly through phosphorylation by CDK5/GSK-3β (cyclin-dependent kinase 5/glycogen synthase kinase-3β) or Rho kinase [48,49]. In turn, stimulation of growing axons by Sema3A (semaphorin-3A) activates CDK5 [50]. The gene encoding CMRP2 presents two alternative splicing isoforms that differ in their N-terminus: CRMP2B and CRMP2A, a ~100 amino acids longer isoform. The latter was localized in axons rather than dendrites [51,52] and is likely modulated by conformational changes [53]. Balastik and co-authors contributed to the comprehension of this tightly regulated pathway, demonstrating that Pin1 binds and stabilizes CRMP2A. They observed that Pin1 is driven towards the growth cone after stimulation with Sema3A both in vitro and in vivo conditions [34]. Using a proteomic approach, combining GST-pull down followed by SDS-PAGE and tandem mass spectrometry (LC-MS/MS) experiments, they were the first to identify CRMP2A as major target of Pin1 in postnatal neurons. Pin1 stabilized CRMP2A previously phosphorylated by CDK5 selectively in distal axons [34]. Then, they found reduced level of CRMP2A at the growth cone in different experimental models: primary cortical neurons derived from Pin1 KO mice, cell lines knocked down (KD) for Pin1, or after using the specific Pin1 inhibitor (Juglone). In these experiments, Pin1 KO neurons had significantly shorter axons and this phenotype was completely reverted by over expression of CRMP2A. Therefore, they provided robust evidence on the relationship between Pin1 and CRMP2A [34]. 

Treatment with different concentrations of Sema-3A, and not with lysophosphatidic acid (LPA), a bioactive phospholipid in the Rho-kinase pathway, induced CRMP2 signaling via phosphorylation [54] and collapse of the growth cone both in WT and Pin1 KO primary dorsal root ganglia (DRG) neurons. This observation was associated with a change of Pin1 level and distribution in the growth cone of Pin1 WT. The change was dependent on the stimulation by Sema-3A. These observations confirmed that the catalytic action of Pin1 is specific for the Sema-3A signaling pathway in the vicinity of the growth cone where it co-localizes with CRMP2A [34]. Balastik et al. reported also that Pin1 KO embryos present selective defects of the axon growth that affect several regions of peripheral and CNS, like stunted neurite process and lack of arborization, probably owing to the impaired CRMP2A signaling [55,56]. The authors hypothesized that this uneven neuronal phenotype characterized by axonal defects might be due to compensatory mechanisms put in place for Pin1 deficiency by other members of the CRMP family to rescue reduction of CRMP2A. Indeed, Pin1 expression levels were negatively correlated with susceptibility to neurofibrillary degeneration in different regions of mouse and human brain [34,44].

To confirm in vivo the interplay between Pin1 and Sema-3A signaling, Balastick et al. used a zebrafish model of motor neuron development. Silencing of Sema-3A signaling by using KD of Neuropilin1 (NRP1) induced defects of the motor neuron growth [57,58]. Defects of the motor neuron growth were partially rescued in the simultaneous KD of NRP1 and Pin1. In Pin1 KD silencing of Sema-3A (NRP1 KD) produced a milder phenotype of motor neuron growth defects owing to the reduced stabilization of phosphorylated CRMP2A by Pin1. This would further support the notion that Pin1 regulates the Sema3A-driven axonal guidance. Indeed, Pin1 stabilizes CMRP2A selectively in distal axons and buffers low-level Sema3A stimulation both in vitro and in vivo [34].

## 4. Role of Pin1 in Neurodegenerative Disorders

The control of mitotic entry and progression is accompanied by the formation of specific phosphoepitopes such as MPM-2 (mitotic protein monoclonal 2), that are formed on Ser or Thr residues next to a Pro residue. Pin1 modulates function and/or dephosphorylation of some of these phosphoproteins, that are mostly recognized by the specific monoclonal antibody MPM-2 [59]. The induction of MPM-2 epitopes is a common feature in a number of neurodegenerative disorders (i.e., AD, FTD with Parkinsonism, progressive supranuclear palsy, corticobasal degeneration, Down’s syndrome, and Pick’s disease) [44]. Therefore, the presence of MPM-2 epitopes suggests the likely involvement of Pin1’s catalytic activity in the pathogenesis of these heterogeneous conditions.

### 4.1. Huntington’s Disease

Pin1 was reported to contribute also to the neurodegeneration seen in a mouse model of Huntington’s disease. The expression of mutant Huntingtin (mHtt) determined the phosphorylation of p53 on a Ser46 residue that made it a target site for binding and modulation by Pin1 [60]. The authors hypothesized that this interaction caused the dissociation of p53 from the apoptosis inhibitor iASPP, promoting the p53 activation cascade in striatal neurons. The authors demonstrated that inhibition of Pin1, by using the specific inhibitor PiB, protected in vitro neuronal cells from mHtt-induced apoptosis. Therefore, inhibition of Pin1 might represent a therapeutic target for the treatment of Huntington’s disease [60]. 

### 4.2. Parkinson’s Disease

Lewy bodies (LBs) are aggregates of proteins that represent the histological hallmark of the Parkinson’s disease (PD). α-synuclein, a presynaptic neuronal protein of unknown function, is the major constituent of LBs. Post-mortem histochemical analysis of patients’ brain revealed the detection of Pin1 in the 50–60% of LBs [61]. Pin1 interacted indirectly and co-localized with α-synuclein in intracellular inclusions. Indeed, Pin1 bound the phosphorylated form of synphilin-1 mediated by casein kinase II (CKII) (player in cell cycle progression) and modulated the interaction between α-synuclein and synphilin-1. In neurons from substantia nigra or locus ceruleus of patients with PD, Pin1 had higher affinity for α-synuclein-synphilin-1 complex than for tau protein (in patients with AD it has the opposite affinity) and did not co-localize with the latter in LBs. Again, we face an example of the diverse modulation of Pin1 depending upon cellular types and biological contexts [61].

### 4.3. Temporal Lobe Epilepsy

**Figure 2:**
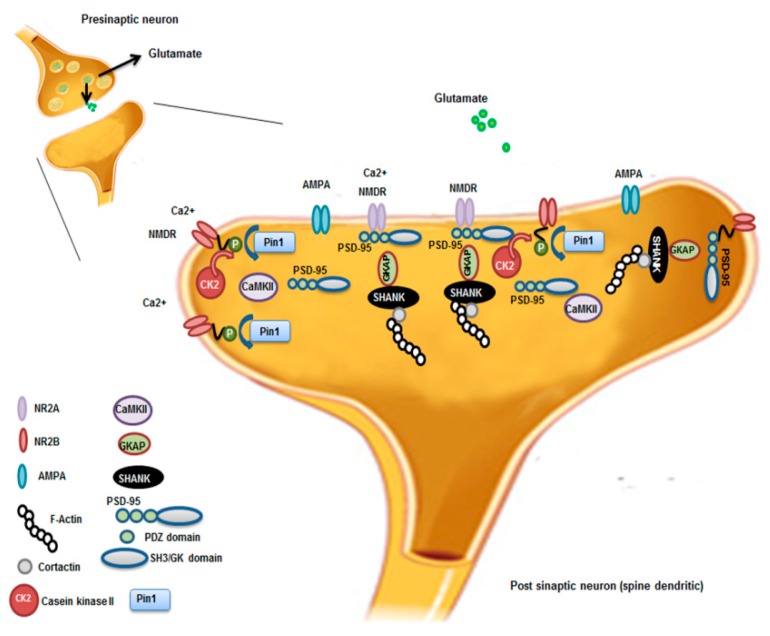
Pin1 regulation of NMDAR complex and synaptic plasticity. Synaptic activation of NMDAR stimulates Ca2^+^/Calmodulin-dependent protein Kinase II (CaMKII) and casein kinase II (CK2) activity. CK2 phosphorylates the PDZ (postsynaptic density-95(PSD-95)/Discs large/zona occludens-1) ligand of NR2B. Pin1 binds and stabilizes probably the conformational change of NR2B phosphorylated disrupting the interaction between NMDAR on the cell surface and the PDZ domains of PSD-95. This leads to destabilization and internalization of surface NMDAR that influences, in turn, synaptic transmission and spine morphology. NR2A; NR2B; AMPA; F-Actin; Contarctin; CaMKII, Ca2^+^-Calmodulin dependent protein Kinase II; CK2, casein Kinase 2; PSD-95 presents three PDZ domains, SH3 (Sarc homology 3 domain) and GK (guanylate kinase-like domain) domains; Pin1; GKAP, Guanylate kinase associate protein; Shank, shank protein.

In epileptic mice and patients with TLE, Pin1 was down-regulated as well, suggesting the involvement of the protein also in this disease [62]. Pin1 modulates an important neuronal protein, gephyrin [63], a postsynaptic scaffolding protein that favors the clustering of GABA(A) receptors at inhibitory synapses and that is down expressed in TLE patients [64]. Based on immunofluorescence, Pin1 localized in cytoplasm and cytoplasmic membranes of neurons from hippocampus and neocortex of epileptic patients and pilocarpine-induced epileptic mice [62]. Pin1 expression was down regulated in the hippocampus and cortex of mice with spontaneous recurrent seizures (SRS), compared to controls following the epileptic seizures. The authors performed immunoprecipitation experiments that demonstrated the interaction of Pin1 with NMDAR subunits 2A/2B (NR2A/2B) containing NMDA receptors (NMDARs) and not α-amino-3-hydroxy-5-methyl-4-isoxazole propionic acid receptors (AMPARs). The authors speculated that Pin1 influenced competitively the formation of synapse-associated protein-95/NR2B (PSD95/NR2B) complex thus, negatively affecting NMDAR-mediated synaptic transmission and spine morphology. The reduced expression of Pin1 caused the decreased surface trafficking of NMDARs by promoting NMDARs internalization with the net result of reduced neuronal hyper-excitability. Nevertheless, the fine mechanism by which Pin1 modulates NMDARs internalization remains unclear (Figure 2).

In this disease too, Pin1 with its modulation might represent a target to modulate neuronal hyper-excitability [62].

### 4.4. Pin1 and Alzheimer’s Diseases (AD)

AD is the most frequent form of dementia in the elderly causing progressive cognitive decline and memory loss. AD is characterized by widespread apoptosis of neurons and increased deposition of extracellular plaques and intracellular neurofibrillary tangles (NFTs) within the brain. NFTs are aggregates of microtubules that result from the hyperphosphorylation of tau protein. They are markers of AD: the amount of NFT deposits is correlated with the degree of neurodegeneration [65]. The plaques are primarily comprised of aggregated amyloid-β-peptide (Aβ) derived from the increased processing of the amyloid precursor protein (APP) [66]. 

Elderly Pin1 KO mice develop a complex phenotype of premature aging characteristics: namely, reduced body size, telomere instability, decreased germ-cell proliferation rate, cognitive impairment and neuronal degeneration that resembles human AD [44,67,68,69,70]. Of note, Pin1 KO mice show anomalous behavior and enhanced tau accumulation in the brain. These evidences support the argument that Pin1 dysfunction/deficiency is a pivotal determinant of the AD progression [3]. 

Level of Pin1 expression increased during neuronal differentiation and remained high during the lifespan [14,44]. In neuronal cells of AD patients, Pin1 was downregulated and the degree of downregulation was inversely correlated with the neuronal loss, since loss the protective ability of the enzyme against degeneration [44]. Pin1 regulated the *cis*/*trans* conformational changes of tau and APP proteins after their phosphorylation by kinases, such as GSK-3β [71]. Pin1 catalyzed the conformational switch of tau and APP proteins from the dysfunctional *cis*-toward the functional *trans*-structure. As consequence, tau could be dephosphorylated and degraded [39,70,71]. The accumulation of phosphorylated tau in Thr231-Pro motif was an early event in patients affected by mild cognitive impairment (MCI) [71]. In fact, Pin1 co-localized with phosphorylated tau and modulated the assembly of tau and tubulin into microtubules [72]. Pastorino and co-workers demonstrated that Pin1 drove the processing of APP and the formation of nonamyloidogenic βamyloid (Aβ) plaques. Indeed, the over-expression of Pin1 reduced Aβ secretion from cell cultures [70]. 

Pin1 modulated the APP processing toward the healthy nonamyloidogenic form by catalyzing *cis*-to *trans*-isomerization of the phosphorylated Thr668-Pro motif of APP [70] both directly and inhibiting the phosphorylation of APP in that motif induced by GSK-3β activity [73]. 

Pin1 inhibited GSK-3β activity trough the binding to a phosphorylated Thr330-Pro motif and catalyzing substrate isomerization [73]. This became evident in H4 neuroglioma cells transfected with WT or mutated construct GSK-3β T330A that did not affect the basal activity of the enzyme. In H4 cells, transfected with the mutated T330A GSK-3β, Pin1 was not able to bind and inhibit GSK-3β activity, thereby increasing the level of toxic amyloidogenic APP processing [73]. It has been speculated that the initial steps of tau phosphorylation have a neuronal protective role against the toxicity exerted by Aβ deposits [74,75], but they become detrimental when the excessive phosphorylation of tau causes the accumulation of NFTs [75]. Thus, the fine regulation of this post-translational modification is central in the pathogenesis of AD.

Alternatively, Pin1 may modulate APP processing to inhibit the phosphorylation of Thr668-Pro motif of APP performed by GSK-3β [73,76] Processing was observed in Pin1 WT or KD cells transfected both with WT or the mutated T330A GSK-3β constructs [73]. When Pin1 KD cells were transfected with both WT and mutated T330A GSK-3β, they showed comparable levels of pThr668, while Pin1 WT cells showed reduced levels of pThr668 only when transfected with WT GSK-3β construct. Therefore, the presence of Pin1 was needed for APP degradation following its binding to the Thr330-Pro motif of GSK-3β, resulting in its inhibition that then reduced phosphorylation of Thr668 [73]. 

In a molecular model, Pin1 was critical to regulate the active and stable conformation of Akt protein [77], that is an important player in cell survival, growth, migration and proliferation. The Akt signaling cascade is one of the survival pathways activated by neurotrophins through the binding to the tyrosine kinase (Trk) family of receptors. In *post mortem* studies of AD brains, Pin1 and neurotrophins expression levels were reduced in parallel [14]. As Angelucci and Hort hypothesized, another way for Pin1 to determine the fate of neuronal cell survival/death in neurodegenerative disorders might be related to the balance between expression of Pin1 and of neurotrophins (i.e., the brain derived growth factor, BDNF). Pin1 and neurotrophins can modulate the response of p53 [14,78,79], that acts not only as tumor suppressor, but also as a player in neuronal differentiation process [14,80], toward some transcriptional targets. The authors hypothesized that Pin1, modulating Akt and p53 conformation stability, influenced the signal transduction pathways activated by neurotrophins through the Trk receptors binding [14]. In neurodegenerative conditions, the reduced Pin1 expression level determined the reduced activity of neurotrophins-Trk signal transduction pathway causing an increased induction by p53 of neuronal death [14]. 

Furthermore, Pin1 influenced synaptic plasticity by regulating protein ubiquitination and degradation of postsynaptic density (PSD) proteins. By using a proteomic approach, Xu and collaborators demonstrated the association between Pin1 and Shank proteins at dendritic rafts of neuronal cells and PSD isolated from the synaptosome fractions obtained from the frontal cortical tissues of AD patients and controls [81]. Shank proteins have an “organizing role” in dendritic rafts and PSD. In cultured cortical neurons from Pin1 KO and control mice, Xu and collaborators verified the role of Pin1 in synaptic plasticity, inhibiting both Pin1 (with PiB treatment) and proteasome activity (by using MG132, carbobenzoxy-Leu-Leu-leucinal peptide). Ubiquitinated proteins in the PSD increased after the simultaneous treatment with PiB and MG132. This deregulated pathway determined an enhanced degradation of Shank3 and other PSD proteins, with a consequent alteration in PSD structure from AD brains. Pin1 modulated the NMDA receptor-mediated turnover of Shank proteins. In this regard, Tang and co-workers who studied Pin1 modulation in TLE patients [62], hypothesized that reduced Pin1 activity caused not only misfolded proteins, but also the generation of aberrant synapses contributing to the progression of pre-clinical AD [81]. 

Despite the role of Pin1 in the pathogenesis of AD, none of the PIN1 polymorphisms have been conclusively associated with delayed AD onset [26,65].

## 5. Pin1 Links Brain Impaired Glucose Metabolism and Neuronal Degeneration in AD

We speculate that post-translational modulation of Pin1 links AD among neurodegenerative disorders with peripheral and brain impaired glucose metabolism. Pin1 KO mice developed neuronal characteristics of premature aging similar to those observed in human AD i.e., age-related cognitive decline [44,68,69,70], but also peripheral (in both liver and muscle) and brain impairment of glucose metabolism and altered insulin signaling that resulted in overt glucose intolerance [3]. 

Autopsy studies in AD brains and intracerebral injected mice with streptozocin (STZ) demonstrated that brain insulin resistance and impaired insulin signaling occur early in the natural history of the disease, even before main clinical and histological characteristics develop. Main features in AD brains and STZ treated mice were cognitive impairment, structural deficits of neuronal cytoskeleton, loss of synaptic connections and increased neuronal apoptosis. Therefore, AD emerged as metabolic brain disease characterized by neuroinflammation and impaired energetic metabolism that lead to neuronal damage [16].

### 5.1. Type 3 Diabetes

The presence of defective insulin signaling and reduced glucose intake in the brain might be therefore early features that precede the diagnosis of overt cognitive deficits [16]. 

In *post mortem* brains (i.e., cerebral cortex, hippocampus and hypothalamus), the expression levels of insulin, insulin-like growth factor (IGF) 1, and IGF1 receptors were in AD patients significantly lower than in healthy age-matched individuals [82]. mRNA expression of IGF-2 tended to be different as well. Expression levels were correlated with the degree of cognitive impairment (Braak stages) in AD patients. Differential regional distribution of insulin, IGFs and their receptors was confirmed in primary neuronal cultures from rat fetal brains. Impairment of brain insulin signaling was induced in fetal rat by gestational exposure to ethanol. In this model, there was reduced production of insulin and IGFs as well as reduced expression of their receptors in different areas of the CNS. Reduced synthesis of growth factors in the brain could account for the increased neuronal death. Steen and de la Monte hypothesized that brain insulin resistance manifested as the inability to compensate for reduced secretion of insulin and IGFs with overexpression of their receptors [82]. They speculated a new type of diabetes that they named “type 3 diabetes” [16,17]. T3D would be characteristic of AD patients, affecting glucose metabolism exclusively in the brain or more broadly [75]. The reduced expression of insulin and IGF-1 receptors might explain the insulin resistance registered in the AD brain, thereby affecting negatively the insulin transduction pathway. Insulin/IGF-1 signaling upon binding to receptors activates the auto phosphorylation of receptors at Tyr residues and the activation of docking proteins, named insulin receptor substrates (IRS-1-4) and responsible for signal transduction. The intracellular signal determines the activation of mitogenic functions through MAPK/extracellular signal-regulated kinase (MAPK/ERK) and metabolic functions through the phosphatidyl-inositol-3-kinase/Akt (PI3K/Akt) pathway that phosphorylates and inactivates GSK-3β. Given the impaired insulin/IGF-1 signal transduction, the expression level of IRS-1 was reduced in tissues from AD brains with respect to controls [82]. As consequence, the decrease of phospho-Akt and the increase of active form of GSK-3β, responsible for tau hyperphosphorylation, increased the apoptotic stimulus and caused mitochondrial dysfunction, thereby increasing mitochondrial-mediated apoptosis and oxidative stress [82]. 

Insulin and IGFs levels were reduced in brain tissues of AD patients and this was associated with the reduced synthesis of tau [82,83]. Growth factors stimulated tau protein expression in neuroblastoma cells [83]. The condition of brain insulin resistance was characterized by reduced levels of insulin and IGFs, no compensatory hyper-expression of their receptors and therefore a reduced transduction pathway [16,17,82]. de la Monte and collaborators speculated that the brain insulin resistance accounts for most of the molecular, histological and biochemical damages found in AD patients and develops before the onset of the clinical AD phenotype [17].

Therefore, insulin resistance that manifests in patients with T2D as peripheral hyperinsulinemia and increased release of IGF1, defective binding to receptors, impaired insulin signaling in muscle, adipose tissue and liver, could also be characterized in the brain by reduced release of insulin and IGFs and results as well in impaired insulin signaling in the brain. In both cases, impaired signaling causes reduced glucose utilization as determined by clamp and positron emission tomography studies. To further support commonalities in the pathogenesis of T2D and T3D, there is evidence from clinical trials using anti-diabetic dugs (namely analogs of glucagon like peptide 1, GLP1) in patients with T2D and/or AD that demonstrate the amelioration of the cognitive performance in parallel with the improvement of the glucose metabolism [17]. 

### 5.2. Pin1 and Insulin Pathways 

Pin1 modulated peripheral glucose metabolism influencing independently both insulin secretion and sensitivity. β-cell-specific Pin1 KO (βPin1 KO) mice [84] developed glucose intolerance owing to reduced insulin secretion but preserved peripheral insulin sensitivity. βPin1 KO mice presented reduced β-cell mass as compared to controls suggesting that Pin1 affects β-cells proliferation. Cultured Pin1 KO β-cells had reduced intracellular Ca2^+^ response to glucose- and KCl, despite preserved cellular ATP and insulin content. The mechanism by which Pin 1 influenced Ca2^+^ response implied salt inducible kinase 2 (SIK2) and p35 protein. Pin1 interacted with SIK2, a protein belonging to the AMP-activated protein kinase (AMPK) family that is a key player for insulin secretion. The binding of Pin1 with SIK2 enhanced SIK2 kinase activity that, in turn, promoted p35 protein degradation and down-regulation of the p35-CDK5 (Cyclin-dependent kinase-5) complex activity, a negative regulator of Ca2^+^ influx [84].

Experiments in KO mice, primary human endothelial cells and peripheral blood monocytes (PBMCs) of T2D patients demonstrated that hyperglycemia caused Pin1 upregulation and the latter, in turn, mediated vascular-damage occurring in diabetic patients [85]. Hyperglycemia was associated with reduced methylation of the *Pin1* gene promoter [85], and that is another finding commonly observed also in patients with AD. Indeed, Arosio and collaborators found upregulated *Pin1* gene expression in PBMCs of late onset AD patients and significantly reduced percentage methylation of *Pin1* gene promoter. In PBMCs of these patients, Pin1 quantity and activity tended to be reduced as well [86]. To explain this apparent divergent behavior, Wang [87] suggested that Pin1 reduction contributes first to the accumulation of hyperphosphorylated tau in AD patients, but successively Pin1 is over expressed to compensate for the increased formation of Aβ plaques.

AMPK is the major sensor for cellular energy status. Phosphorylation/dephosphorylation cycles of the α subunit control the activation state of AMPK. In presence of nutrients deficiency, the AMP/ADP molecules bind CBS domain of the γ subunit determining a conformational change of AMPK that protects the α subunit from dephosphorylation by protein-phosphatase 2C (PP2C). Pin1 regulated negatively AMPK, binding its CBS domain in the γ subunit. By doing so, it inhibited the binding of AMP/ADP to the γ subunit of AMPK. Therefore, Pin1 KO mice were resistant to high-fat-diet (HFD) induced obesity [88,89], with the activity level of AMPK in muscle significantly higher than in WT mice [88]. In that interaction with AMPK, Pin1 may represent a therapeutic target also to treat obesity and diabetes. 

Indirect evidence in diabetic db/db treated by GLP1-analog demonstrated that PIN1 modulates insulin signaling also at the central level [72,90], being both an insulin receptor substrate-1 (IRS-1) and Akt binding partner [77,89]. About the role of Pin1 on IRS-1 signaling cascades, two hypotheses were formulated. Pin1 isomerase-activity may favor IRS-1 phosphorylation [91], or alternatively the isomerization of IRS-1 may prevent its dephosphorylation by the protein Tyr phosphatase [3]. Although Pin1 interacted with IRS-2, it did not influence its phosphorylation, likely because of a less efficient binding [89,92]. Pin1 isomerization activity induced a reduced response to insulin (insulin resistance), as demonstrated by its association with stress-induced c-Jun N-terminal kinase (JNK) and/or ribosomal protein S6 kinase (S6K), through the modulation of Ser-phosphorylation of IRSs [3,69,93]. In particular, Pin1 increased JNK kinase activity and the phosphorylation level of S6K. In HFD conditions, the Pin1 expression level increased determining the hyper-activation of JNK and S6K. Thus, in this metabolic condition the positive effect of Pin1 on IRS-1 and insulin cascade was abolished causing, instead insulin resistance.

**Figure 3:**
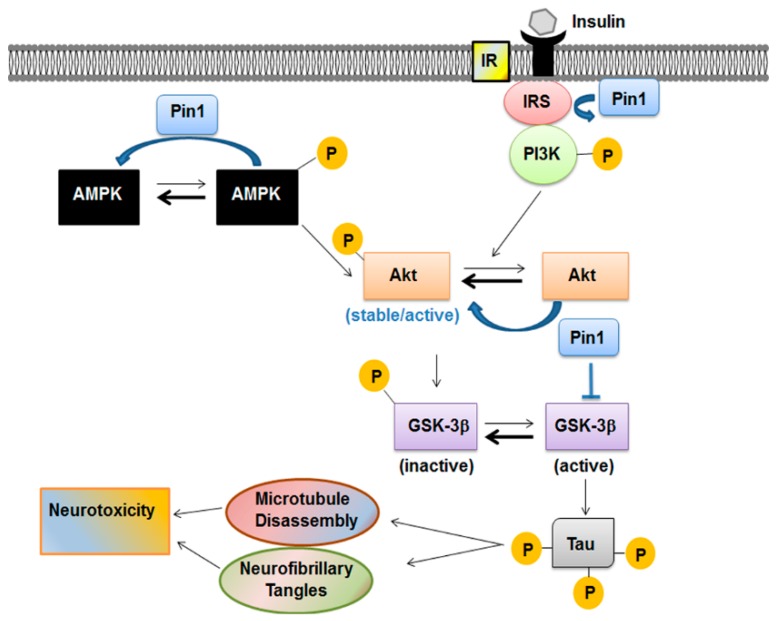
Pin1 in the metabolic pathway acts as a negative regulator of AMPK and modulates the Akt active form. Insulin promotes neuronal cytoskeletal dynamics via Akt phosphorylation. Activation of insulin signaling leads usually to activation of phosphatidyl-inositol-3-kinase/Akt (PI3K/Akt) pathway. After being activated through its phosphorylation mainly at Thr308 residue, Akt phosphorylates glycogen synthase kinase-3β (GSK-3β) at Ser9 and inactivates GSK-3β kinase activity. GSK-3β is also the major protein kinase regulating tau phosphorylation in the brain. Pin1 modulates all these players involved in the signaling cascade.

Together with upstream kinases and protein phosphatases, Pin1 influenced the equilibrium between active and stable Akt versus the inactive and unstable form [77]. This modulation represents another way for Pin1 to influence the insulin transduction signaling. In fact, insulin promoted neuronal cytoskeletal dynamics via Akt phosphorylation [94]. Activation of insulin signaling leads usually to the activation of PI3K/Akt pathway. Akt, after being activated through its phosphorylation mainly at Thr308 residue, phosphorylates GSK-3β at Ser9 and inactivates GSK-3β kinase activity [95]. GSK-3β is one of the first enzymes involved in the regulation of glycogen synthase but is also the major protein kinase regulating tau phosphorylation in the brain [96,97] (Figure 3). GSK-3β is inactivated if phosphorylated in Ser9 residue by PI3K/Akt pathway. Its activity was found to be increased in insulin resistance conditions, suggesting that this kinase is pivotal in the regulation of peripheral and brain glucose utilization [75].

### 5.3. Pin1 and GSK-3β(Glycogen Synthase Kinase-3β) Modulation

Hence, Pin1 modulates insulin signaling by acting on Akt [77] and GSK-3β [73] activities. In brain extracts of elderly diabetic db/db mice, Akt activity was down-regulated and GSK-3β activity increased leading to the enhanced phosphorylation of tau protein and formation of NFTs [90]. It may be speculated that, more generally, the oxidative damage occurring in the diabetic brain results in impaired phosphorylation of tau protein and Pin1 modulation is implied. Oxidative modification of Pin1 at Cys113 residue was associated with reduced catalytic activity and expression in hippocampus of patients with MCI and AD too [72,98,99,100]. 

The mutual interaction between GSK-3β and Pin1 become evident also in experimental models of hereditary hemochromatosis, whereas homozygous patients carrying the HFE hemocromatosis mutation have enhanced risk of diabetes (“bronze diabetes”) if untreated. In SH-SY5Y cells carrying H63D mutation of the HFE gene and in H67D transgenic mice, phosphorylation of Pin1 on Ser16 was increased and resulted in decreased enzyme activity. The treatment with Trolox, an antioxidant, rescued Pin1 activity in both models [101]. In the in vitro model, tau phosphorylation was increased paralleling GSK-3β activity [99], while Pin1 activity was consequently reduced [101]. Conversely, the inhibition of GSK-3β activity by lithium was associated with the increase of Pin1 activity, reinforcing the notion that GSK-3β and Pin1 interact with each other’ to influence the ability of Pin1 to modulate its substrates such as tau protein [101,102]. 

## 6. Conclusions

The prolyl isomerase Pin1 plays a central role in the switching of proline-directed phosphorylation signaling: it induces the isomerization of the *cis*/*trans* configuration of protein substrates that are phosphorylated. As molecular “switch” that turns on or off enzyme activities or entire signaling pathways, Pin1 modulates many cellular functions both in physiological processes and pathological conditions (i.e., cancer, neurodegenerative and metabolic diseases). Pin1 exerts different and even opposite effects in vivo that are cell specific, depend upon specific protein substrates and are associated with phosphorylation of distinctive Pin1 binding sites. 

Among the various regulatory functions, Pin1 controls neuronal differentiation during brain development by stabilizing β-catenin and synaptic plasticity/degradation of postsynaptic density proteins. Pin1 protects against oxidative damage of neurons, thereby protecting them from neurodegeneration. Indeed, impaired Pin1 function is associated with neurodegeneration and cognitive dysfunction in patients with AD. But Pin1 is also a master regulator of neuronal energy metabolism and interferes particularly with the cell insulin signaling. Because of these dual major roles on neuroprotection and metabolism, we and others speculate that impaired Pin1 function is one of the pivotal molecular links between neurodegeneration and impaired glucose metabolism, both manifesting in patients with dementia. Therefore, we report evidence supporting this hypothesis. There is large overlap between Pin1 and insulin signaling pathways within the brain, with Pin1 influencing insulin signaling. 

Therefore, Pin1 may also represent a treatment target to prevent the onset of brain impaired glucose metabolism that is deemed by some researchers as one of the first hints in the pathogenesis of the AD. It may be effective to prevent the onset of or delay the neurodegenerative process, since acting as isomerase, Pin1 catalyzes the conformational switch of tau and APP proteins from the dysfunctional *cis*-toward the functional *trans*-structure and enhances tau dephosphorylation and degradation.
